# Non-equilibrium states and interactions in the topological insulator and topological crystalline insulator phases of NaCd_4_As_3_

**DOI:** 10.1063/4.0000273

**Published:** 2025-02-05

**Authors:** Tika R Kafle, Yingchao Zhang, Yi-yan Wang, Xun Shi, Na Li, Richa Sapkota, Jeremy Thurston, Wenjing You, Shunye Gao, Qingxin Dong, Kai Rossnagel, Gen-Fu Chen, James Freericks, Henry C Kapteyn, Margaret M Murnane

**Affiliations:** 1 Department of Physics and JILA, University of Colorado and NIST, Boulder, Colorado 80309, USA; 2 Institute of Physics and Beijing National Laboratory for Condensed Matter Physics, CAS, Beijing, China; 3 Institute of Experimental and Applied Physics, Kiel University, D-24098 Kiel, Germany; 4 Ruprecht Haensel Laboratory, Deutsches Elektronen-Synchrotron DESY, D-22607 Hamburg, Germany; 5 Department of Physics, Georgetown University, Washington, DC 20057, USA; 6 KMLabs Inc., 4775 Walnut Street, #102, Boulder, Colorado 80301, USA

## Abstract

Topological materials are of great interest because they can support metallic edge or surface states that are robust against perturbations, with the potential for technological applications. Here, we experimentally explore the light-induced non-equilibrium properties of two distinct topological phases in NaCd_4_As_3_: a topological crystalline insulator (TCI) phase and a topological insulator (TI) phase. This material has surface states that are protected by mirror symmetry in the TCI phase at room temperature, while it undergoes a structural phase transition to a TI phase below 200 K. After exciting the TI phase by an ultrafast laser pulse, we observe a leading band edge shift of >150 meV that slowly builds up and reaches a maximum after ∼0.6 ps and that persists for ∼8 ps. The slow rise time of the excited electron population and electron temperature suggests that the electronic and structural orders are strongly coupled in this TI phase. It also suggests that the directly excited electronic states and the probed electronic states are weakly coupled. Both couplings are likely due to a partial relaxation of the lattice distortion, which is known to be associated with the TI phase. In contrast, no distinct excited state is observed in the TCI phase immediately or after photoexcitation, which we attribute to the low density of states and phase space available near the Fermi level. Our results show how ultrafast laser excitation can reveal the distinct excited states and interactions in phase-rich topological materials.

## INTRODUCTION

Topological materials can host unique conducting surface and edge states that are robust against disorder, defects, and modification. In addition, they exhibit interesting surface properties such as spin polarization, momentum locked surface spin,^1,2^ and backscattering suppression,^3^ which makes them promising platforms for technological applications. Due to these unique properties, the search for candidate topological materials for novel technological applications has significantly increased in the past 15 years.^4^ To date, many 2D and 3D topological insulators (TIs), including semi-metallic TIs, dual topology insulators,^5,6^ and topological crystalline insulators (TCIs), have been theoretically predicted;^4^ however, only a few of them have been experimentally realized.

TCIs are a new class of quantum materials in which the surface band topology is protected by crystal space group symmetries,^7–10^ unlike time reversal symmetry in a non-trivial Z2 topological insulator (TI).^8,9,11,12^ In general, these materials possess a bulk bandgap with the coexistence of bulk-boundary gapless Dirac-cone-like surface states. Owing to the rich variety of crystalline symmetries (230 crystalline space groups),^13^ many TCIs have been identified with surface states protected by mirror symmetries, glide mirror symmetries, and more recently rotational protected symmetries.^14^ In addition, these materials undergo topological phase transitions by tuning the composition, concentration, temperature,^15,16^ and pressure.^17–19^ Being phase rich materials, their non-equilibrium states might exhibit many-body physics related to electrons, spins, phonons, polarization, bulk-surface carrier dynamics, and carrier relaxation channels, which have been probed by photoemission^20–23^ and optical techniques.^24–26^ However, only a few studies to date have explored the non-equilibrium states and interactions in these materials, which can also reveal novel information about the ground state interactions.

Time- and angle-resolved photoemission spectroscopy (tr-ARPES) is a powerful method for probing the dynamic electronic order of quantum materials with energy and momentum resolution. This makes it possible to track surface and bulk carrier dynamics, as well as the coherent many-body interactions between charges, phonons, and spins.^27–29^ Most studies to date have probed Z2 TI materials, and in particular, group IV-VI compounds and their derivatives involving alloys that lead to a TCI or TI, as well as prototype TI compounds containing Bi.

Here, we study the ultrafast response and relaxation of NaCd_4_As_3_, a material that exhibits dual topology—a topological crystalline insulator phase at room temperature and a topological insulator phase at temperatures below ∼190 K. In the low temperature TI phase, although only weak photoexcitation is observed during the ∼40 fs laser pulse, a very large chemical potential shift of >150 meV within Γ ± 0.15 Å^−1^ near the Fermi level (*E*_F_) is observed, which maximizes after ∼0.6 ps and persists for ∼8 ps. Moreover, the electron temperature (*T*_e_) also maximizes on similar timescales of ∼0.6 ps, which is long after the laser excitation pulse. This behavior and its timescales suggest that the electronic and lattice orders are strongly coupled and that the induced band structure changes are due to partial relaxation of the lattice distortion that is known to be associated with the TI phase (see Fig. 3).^30^ Furthermore, the delayed rise time of electron temperature suggests that the directly excited electronic states and the electronic states near *E*_F_ probed by tr-ARPES are weakly coupled, possibly also due to the partial relaxation of the lattice distortion. The long persistence time of ∼8 ps for the chemical potential (*μ*_F_) shift, excited electron distribution, and enhanced electron temperature are likely related to heat transport from the surface to the bulk, before the ground state TI distortion recovers. In contrast, in the room temperature TCI phase, we observe a very weak excited carrier distribution and minimal chemical potential shift of <30 meV after laser excitation. Our findings are among the first to probe non-equilibrium dynamics and interactions in both the TCI and TI phases of a topological material in its pure form.

## RESULTS

To study the excited state dynamics of NaCd_4_As_3_ in both the TCI and TI phase, a laser pump pulse (1.58 eV photon energy and 40 fs pulse duration) was used to excite the material. The band structure dynamics were then tracked using a 22.1 eV, 15 fs duration, probe pulse, and the resulting photoemitted electrons are analyzed using an angle-resolved photoemission spectroscopy setup (see Materials and Methods section for pump fluence and other details). NaCd_4_As_3_ is an n-type semi-metallic^30,31^ TCI at temperatures above ∼190 K. Below this temperature, it transitions from a monoclinic Cm to a rhombohedral R $\bar{3}$m space group, corresponding to a topological phase transition from a TCI to a TI phase. In the TCI phase, the surface states are protected by the mirror symmetry of the (110) plane at the Г and T points (see S1 of the supplementary material).^51^ Below the transition temperature, the mirror symmetry is broken; however, the band inversion at the Г point persists, as it is protected by time-reversal symmetry. The observed phase transition in our ARPES data before the pump pulse agrees well with previous static ARPES reports^31^ (see S1 of the supplementary material),^51^ within our energy resolution.

**FIG. 1.**
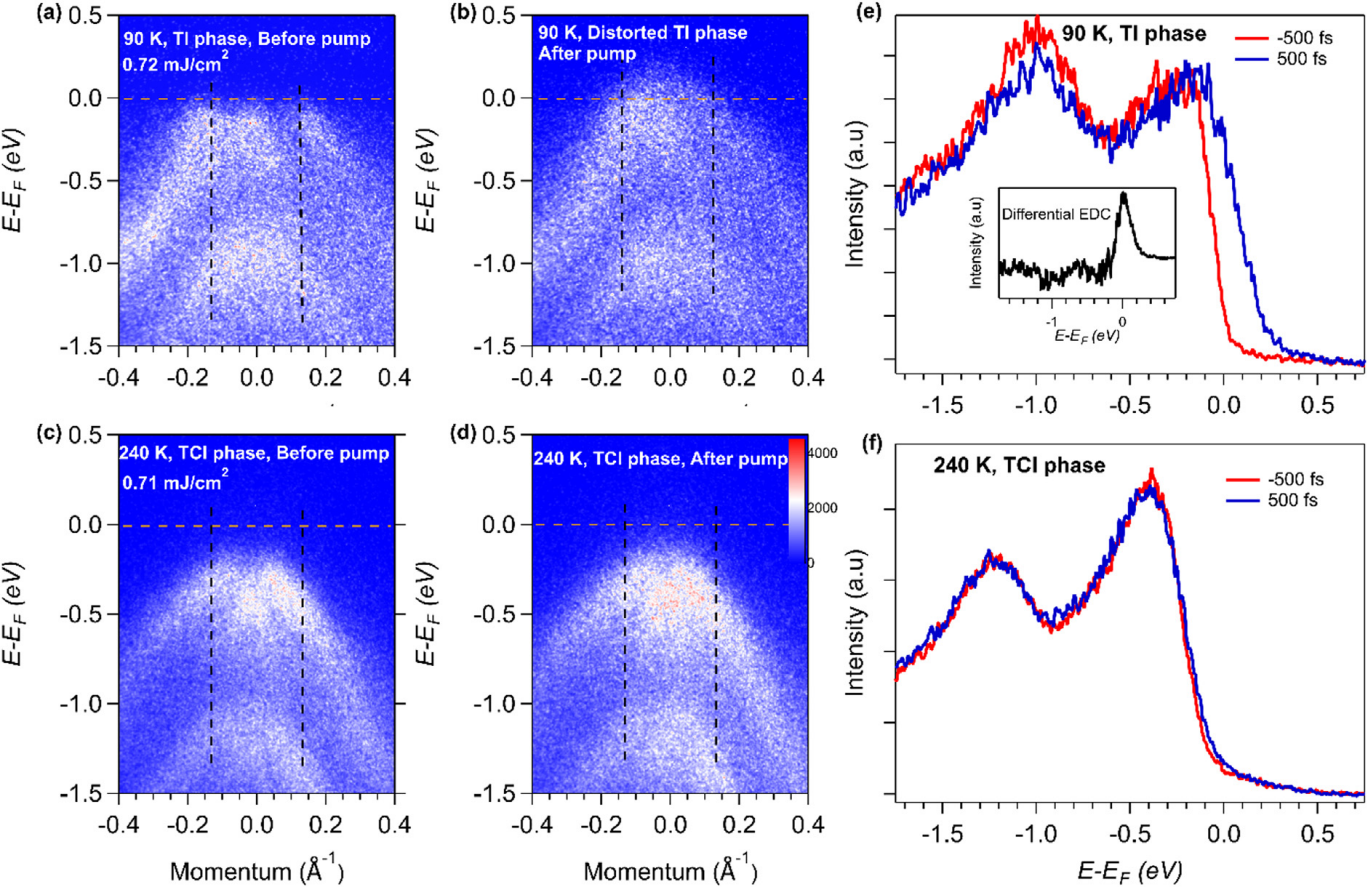
2D ARPES plots of NaCd_4_As_3_ taken at 90 K [(a) and (b)] and 240 K [(c) and (d)]; (a) and (c) before and (b) and (d) after laser excitation. The horizontal dotted brown line indicates the Fermi level. The corresponding energy distribution curves (EDCs), integrated over the momentum range indicated by the vertical dashed line, at 90 and 240 K are shown in (e) and (f), respectively. The inset in (e) is a differential EDC curve before and after laser excitation.

**FIG. 2.**
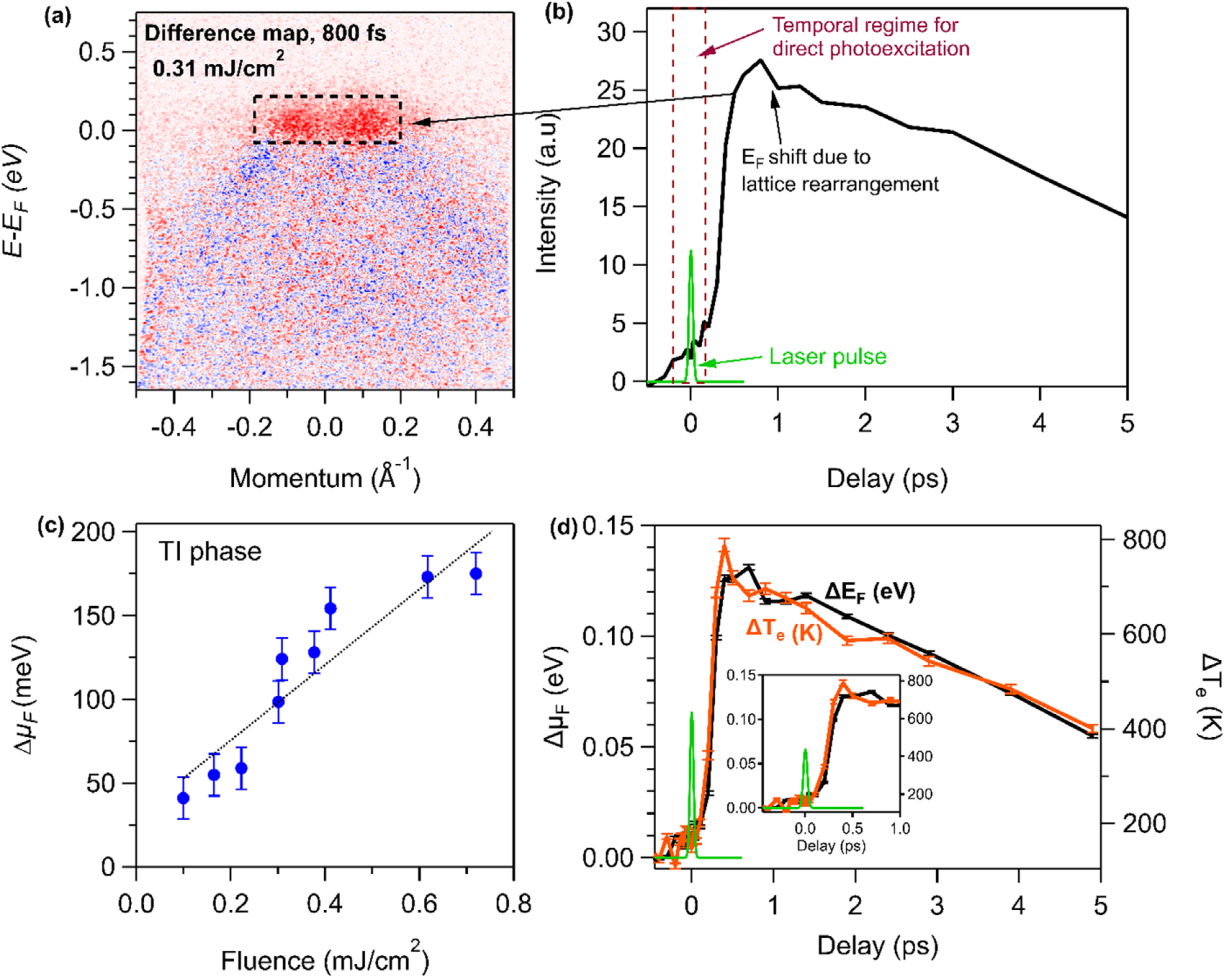
(a) Differential tr-ARPES map before and after laser excitation (800 fs) of the TI phase at 90 K. (b) Electron dynamics integrated over the momentum and energy range indicated by the black dotted box in (a). (c) The maximum change in the chemical potential shift as a function of pump laser fluence. The error bar indicates the Δ*μ*_F_ range obtained by varying momentum integration area centered at Γ to obtain EDCs of interest for data analysis (see S3 of the supplementary material).^51^ (d) Change in electron temperature (Δ*T*_e_) and chemical potential shift (Δ*μ*_F_), both obtained from the Fermi–Dirac distribution.

Typically, in a topological material, bulk bands and surface states (SS) coexist near *E*_F_—in which case electrons would initially be excited from both bulk bands and surface states^28^ into unoccupied states. In the TCI phase as shown in Fig. 1, few excited state carriers are observed after the pump pulse. In contrast, in the low-temperature TI phase, as shown in Figs. 1 and 2, the 40-fs laser pulse initially excites a small population of carriers. Then, a transient state starts to evolve and maximizes on timescales of ∼0.6 ps (Fig. 2), which is far slower than the duration of the laser excitation pulse. The transient state appears to be continuous across *E*_F_, i.e., states above and below *E*_F_ are populated.

To determine the time-dependent band edge (i.e., the chemical potential *μ*_F_) and electron temperature T_e_, energy distribution curves (EDCs), integrated at ∼Г±0.15 Å^−1^ (black dotted vertical lines in Fig. 1), were plotted [see Figs. 1(e) and 1(f) and S2A(a) of the supplementary material].^51^ Then, the instantaneous chemical potential and electron temperature can be extracted by utilizing a Fermi–Dirac (F-D) distribution to fit the energy distribution curve at various delay times (see Sec. S2 of the supplementary material^51^).^20,21^ We note that the Fermi–Dirac fits to the EDC curves used to extract *T*_e_ are of very high quality [see Fig. S2A(a) of the supplementary material], reproducing the detailed experimental data extremely well. In Fig. S2A(a), the change in the position of Fermi level is marked with a dashed blue line; the extracted *μ*_F_ value using this approach is in strong agreement (within the error bars) with an alternative approach to quantify *E*_F_ by determining the center of the Fermi edge (described in Sec. S2 of the supplementary material).^51^ Finally, the observed changes in *μ*_F_ and T_e_ are large, and as expected, certain features in the EDC curves do not change after laser excitation. For example, the intrinsic peak and width associated with the electronic structure of the TI phase around 0.2 eV below *E*_F_ do not change, e.g., the Lorentzian peak position and width labeled E_LP_ (brown vertical dashed line) in S2A(a) of the supplementary material.^51^ Thus, many features in the Fermi–Dirac fit are fixed (e.g., the position of the E_LP_ peak), and a global fit is performed for all time delays to extract T_e_ and *E*_F_. Unphysical models that might vary the position or width of the leading E_LP_ peak gives inconsistent fit results (see Sec. S2 of the supplementary material).^51^

In the TI phase, a large upward energy shift of the leading edge of ∼170 ± 25 meV [see Fig. 2(c) and Sec. S3 of the suppplementary material^51^] was obtained, while this shift is only ∼30 meV in the TCI phase. Figure 2(d) plots the extracted values for Δ*μ*_F_ and Δ*T*_e_ for the TI phase. Both the chemical potential shift and the electron temperature exhibit a slow buildup of ∼0.6 ps and a long persistence time of ∼8 ps, followed by a relaxation of the excited electron distribution and the electron temperature. In particular, the electron temperature increases from the equilibrium value of 90 to ∼800 K at the peak value of Δ*μ*_F_ [see Fig. 2(d)] in the TI phase. This delayed rise time of the electron temperature *T*_e_ is in sharp contrast to the very fast rise time that is observed in most materials probed by tr-ARPES, which peaks during the laser excitation pulse. For the TI phase of NaCd_4_As_3_, the ARPES spectra show no significant excited state population immediately after laser excitation. Hence, the delayed increase in *T*_e_ suggests that the Cd 4*d* and As 4*p* orbitals we probe (near *E*_F_) are different from the orbitals occupied by the hot electrons immediately after excitation. However, after partial relaxation of the lattice distortion in ∼0.6 ps—leading to changes in band structure and orbital occupancy—the ARPES spectra are sensitive to these hot electrons, as shown in Fig. 2(d). They are also sensitive to the polarization of the EUV probe.

**FIG. 3.**
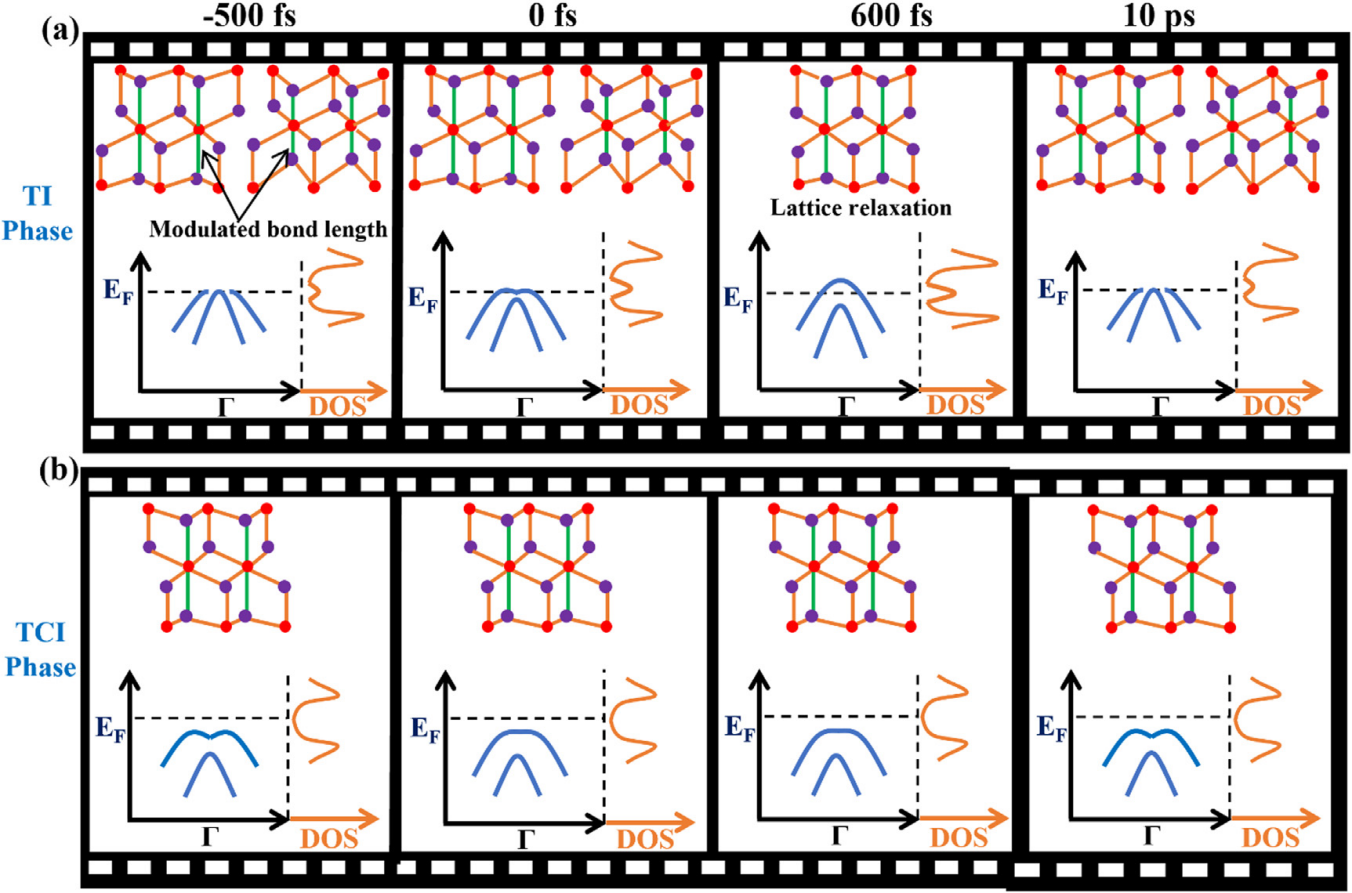
Schematic of the coupled lattice and electronic orders and DOS near the Fermi level of NaCd_4_As_3_ in the (a) TI phase and (b) TCI phase after excitation by an ultrafast laser pulse. These are intended as a guide to explain our findings and are not exact band dispersions and DOS. The actual data are presented in Figs. 1, 2, 4 and in the supplementary material.^51^ The lattice structure shown is a section of the Cd–As substructure (filled circles: purple—Cd and red—As atom). The left panels in (a) and (b) (before laser excitation) highlight that the Cd–As bond length (green line) is modulated in the TI phase, but is unchanged in the TCI phase.^33^ The center panels in (a) plot the proposed lattice rearrangement after laser excitation of the TI phase. For a schematic of the full TCI phase, see S1 of the supplementary material.^51^

To better understand the chemical potential shift in the low-temperature TI phase, we investigated the electronic band structure dynamics, which are plotted in Fig. 2. In Fig. 2(a), we integrated the electron population (black dotted box) above the ground state TI Fermi level and plotted the dynamics in Figs. 2(b) and 2(d). The chemical potential shift builds up slowly, and the peak occurs well after the laser pump pulse [see inset of Fig. 2(d)]. This suggests that the changes we observe may be due to a relaxation of the TI lattice deformation, which modulates both the atomic lattice and electronic band structure, in agreement with a recent report.^32^ A schematic of the predicted lattice bond length modulation^33^ and the observed dynamic electronic structure and density of states after laser excitation are shown in Fig. 3. The variation in the response of two phases stems from the enhanced Cd–As bond length modulation that is associated with the TI phase.^33^ As a result, the TI phase undergoes stronger lattice rearrangement (relaxation) after laser excitation [center panel Fig. 3(a)]. In the TCI phase, the initial Cd–As bond length modulation is very low—hence, there are minimal changes in both the lattice structure and chemical potential [Fig. 3(b)] after laser excitation. To identify the specific changes in the lattice structure, techniques, such as ultrafast x-ray scattering^34^ and/or electron diffraction,^35,36^ would be needed.

The largest bond length modulation of nearly 0.7 Å is predicted to occur for the Cd–As bond [see the first panel of Fig. 3(a), green line] in the ground state of the low-temperature TI phase,^33^ which are the bands we probe with tr-ARPES. Such a modulation is not observed in the TCI phase.^33^ Furthermore, the excited carriers appear around Г along the K-Г-K direction—a full mapping in the Г-K direction shows no clear evidence of direct photoexcitation away from the Г-point. This rules out the explicit involvement of charge transfer processes.

Moreover, the slow rise times (∼600 fs) of both the chemical potential shift and the electron temperature occur on timescales far longer than the 40 fs laser excitation pulse. Processes, such as lattice distortion relaxation or electron scattering from higher excited states, could in theory account for the observed slow rise time.^20,22^ For example, a similar timescale of ∼700 fs has been observed in the TI material Bi_2_Se_3_ for excited electrons to scatter from higher lying states to lower bulk states and/or surface states, followed by a ∼1.7 ps timescale for the electron energy relaxation channel via electron–phonon coupling.^20,22^ However, since higher excited states are not observed in our data, this cannot explain the observed slow rise time.

**FIG. 4.**
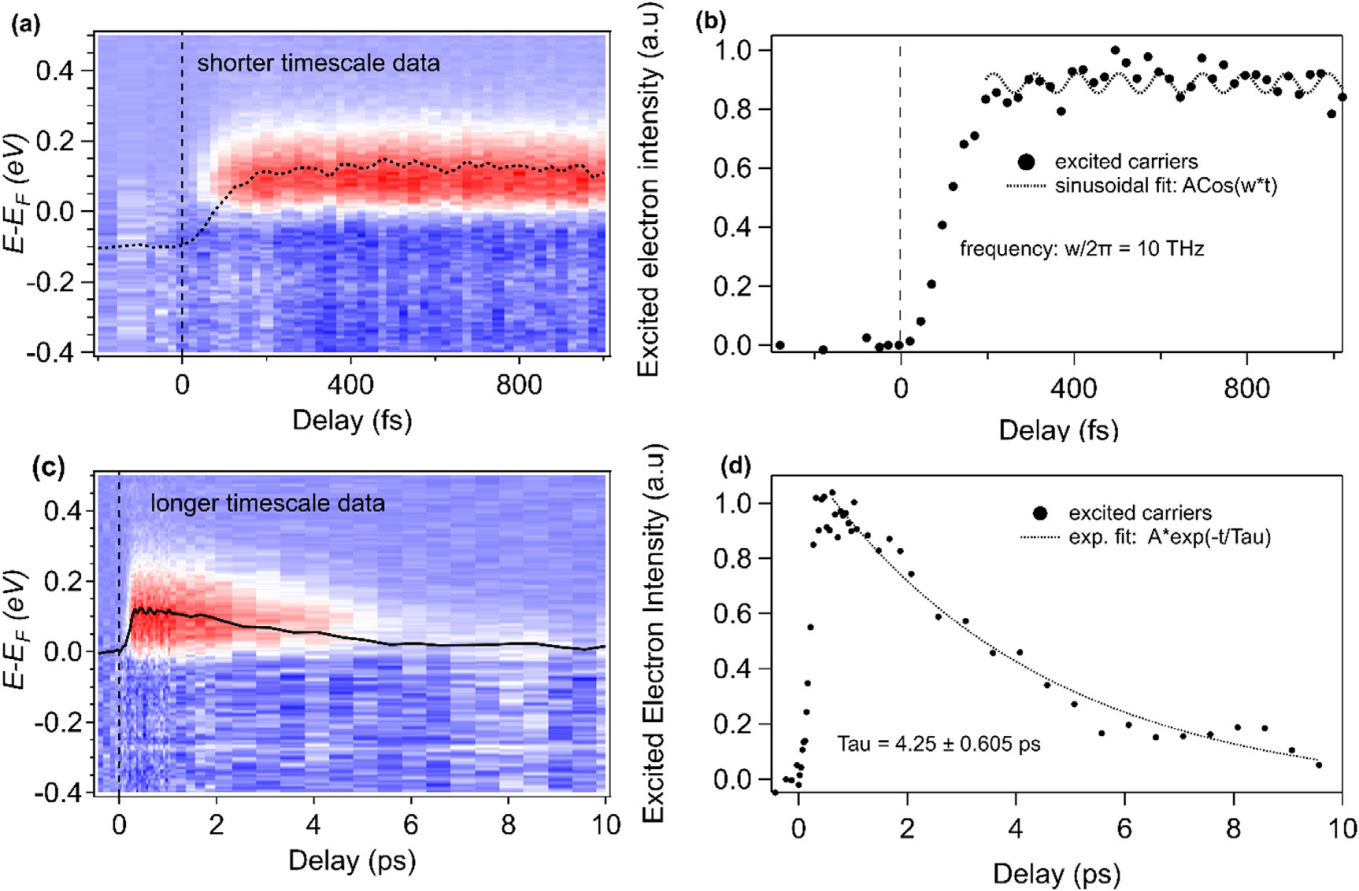
2D temporal map integrated within Г ± 0.15 Å of a differential ARPES spectrum measured at 78 K for (a) a shorter timescale and (c) a longer timescale. The black lines are the 1D temporal dynamics of the same excited electron carriers, which are plotted in (b) and (d), respectively, for better visualization. The dashed lines in (b) and (d) are the sinusoidal and exponential fits, respectively.

Next, we scanned the pump laser fluence to determine if there was evidence of coherent phonon and/or selective phonon mode excitation in the 2D surface states of NaCd_4_As_3_. The chemical potential shift shows a linear dependence on the pump laser fluence, as shown in Fig. 2(c). The maximum Δ*μ*_F_ of ∼170 meV was observed for a laser fluence of 0.72 mJ/cm^2^, which is the maximum fluence that we can use while still avoiding space charge distortion effects. We note that for some datasets (different sample pieces), Δ*μ*_F_ ∼170 meV could be obtained with a slightly lower pump fluence, but further increase in the pump fluence caused spectral distortion. In contrast, at similar laser fluence, a very small chemical potential shift was observed in the TCI phase [see Figs. 1(f) and S3]. We note that NaCd_4_As_3_ has similar absorbed fluence at both of these phases (see the Materials and Methods section). We found a gradual rise in *T*_e_ with pump fluence; however, the rise time is independent of the laser fluence (see S4 of the supplementary material),^51^ further supporting strong electronic and lattice order coupling. The excited state exhibits a single exponential decay with a time constant of 4.25 ps, which is consistent with the timescale observed in most TI materials.^23,29,37^ We note that because of the narrow bulk bandgap of semi-metallic NaCd_4_As_3_, bulk band and surface states coexist near *E*_F_, for both equilibrium and out-of-equilibrium states. Although we do not resolve these states, the dynamics are dominated by the lattice distortion and not by the lifetimes of the excited bulk or surface electronic states. Finally, the long relaxation time of ∼8–10 ps for the material to return to the equilibrium TI state (Fig. 4) indicates that the relaxation mechanism in NaCd_4_As_3_ is likely due to electron–phonon and phonon–phonon relaxation, and heat transport from the surface to the bulk.

There is a hint of weak oscillations (Fig. 4) observed in the excited electron intensity within the first few ps, and a period of 100 fs (frequency of 10 THz) was obtained from the sinusoidal fit. Weak oscillations are also observed in Δ*μ*_F_ and ΔT_e_ obtained from a Fermi–Dirac fit (see Figs. S2 and S5 of the supplementary material).^51^ We speculate that the origin of these oscillations is associated with charge delocalization after excitation of the material, which can excite strongly coupled phonon modes and partially relax the lattice distortion in the TI phase (i.e., the Cd–As bond length modulation). To try to resolve the phonon mode frequency, measurements were taken at different laser fluences—a fast Fourier transform (FFT) indicated a frequency in the 10–14 THz range (see S5 of the supplementary material).^51^ However, due to the constraints of surface degradation of the material over time (see the Materials and Methods section) and the weak nature of the oscillation amplitude itself, it was not possible to take data over a long period to improve the signal-to-noise ratio, hence limiting the signal-to-noise ratio. To precisely determine the phonon modes involved and their frequencies, techniques, such as ultrafast x-ray scattering,^34^ transient reflectivity,^38^ THz or Raman spectroscopies,^33,39–41^ would be required.

## DISCUSSION

There are studies reporting that even small lattice distortions or displacements can induce a topological phase transition.^15,18^ Our data, combined with other recent work,^32^ suggest that strong coupling of the electronic and structural (lattice) orders is responsible for the observed slow rise time of the transient state population, the chemical potential shift, and the increase in electron temperature (Δ*T*_e_), which closely track each other. Such strong couplings have recently been observed in 2D TaSe_2_, where optical excitation excites a high amplitude breathing mode, which in turn modulates the electron temperature, in an isolated isentropic system.^20^ We note that the large time lag (∼0.6 ps) observed for the electron temperature to peak is in contrast to most materials studied to date using ultrafast laser excitation. In most systems, the laser energy is rapidly absorbed by the electrons, so that their temperature peaks within (or soon after) the laser pulse. The very large time lag observed in NaCd_4_As_3_ could arise if electrons from either or both Cd 4*d* and As 4*p* orbitals are initially excited by the laser into delocalized states/orbitals with a symmetry that is not accessed by the EUV probe pulse. Only after the partial relaxation of the lattice distortion and the associated band structure changes, can the hot electrons be probed. Indeed, such sensitivity of orbital bands and their associated odd/even (parity) states to light polarization in photoemission has already been observed.^42–44^ Moreover, the lattice distortion of the TI phase can relax at only slightly elevated temperatures of Δ*T* ∼10 K (far less than what is required to drive an equilibrium TI to TCI phase transition).^45^ The electronic order will follow the evolving structural order, with the rise and fall times for the electron temperature and chemical potential dictated by the lattice distortion relaxation and recovery, as well as cooling via heat transport into the bulk.

Specifically, the transient excited state that lasts for ∼8 ps and that is observed only in the excited TI phase could arise from relaxation of the lattice distortion in the a-b plane of NaCd_4_As_3_ associated with the TI phase. In particular, the atomic bond length between Cd–As along the *c*-axis in the Cd_6_As octahedra within the ${}_{2}^{\infty }[{\text{Cd}}_{4}P_{3}$]^-^ (a 2D polyanionic substructure) has a significant modulation at low temperature—by ± 0.3 Å [see Fig. 1(a), first panel] at 130 K.^30,33^ This bond length modulation shifts the relative distance between the Na^+^ and ${}_{2}^{\infty }[{\text{Cd}}_{4}P_{3}$]^-^ sublattices, causing charge redistribution and hence electronic band structure changes [Figs. 1(a) and 1(c)].^15^ The density of states (DOS), contributed mainly by Cd and As atoms near *E*_F_, increases, providing an increased phase space for excited carriers. Furthermore, the laser-induced reduction in the Cd–As bond length modulation and TI lattice distortion, and hence the coupling of electronic and lattice orders, could flatten the potential energy surface, resulting in a large shift in the chemical potential.

In contrast, the room temperature TCI phase does not exhibit such atomic bond length modulation and has a low DOS near *E*_F_,^30,33^ which limits the transient states accessible in our relatively low laser fluence (0.70 mJ/cm^2^) excitation regime. As noted above, the maximum laser fluence on this sample was limited by space charge effects; thus, it was not possible to directly laser-excite the TI-to-TCI phase transition. Furthermore, we did not observe photoexcited signal immediately after the pump pulse. We speculate that in the TCI phase, the ground state and excited states associated with the Cd–As substructure that we probe are optically dark to our EUV probe. Such optically dark states are determined by the relative phase between the sub-lattices and has recently been reported in a static ARPES study.^46^ Future theoretical studies are needed to validate this hypothesis.

It is likely that multiple bond length modulations that are characteristic of the low-temperature TI phase^33^ are excited as coherent phonon modes after laser excitation.^38,45,47^ However, real-time probing of these excitations would require techniques such as ultrafast x-ray scattering,^34^ transient reflectivity,^38^ or THz spectroscopy.^40–42^ Further dynamical probing with varying photon energy and higher electron energy resolution would be helpful to distinguish the bulk and surface state relaxation channels and to identify competing relaxation pathways. However, we note that the distorted lattice rearrangement in the TI phase will be independent of bulk state and surface states. Thus, as noted above, the measured slow rise time of the excited state and electron temperature could be a consequence of partial lattice relaxation of a distorted TI phase, which is known to be associated with the TI phase (see Fig. 1).^30^ Small lattice rearrangements could introduce electronic structure changes near *E*_F_ while leaving the more deeply bound electronic states intact.

In summary, we have explored the out-of-equilibrium states of NaCd_4_As_3_ in the topological crystalline insulator and topological insulator phases using tr-ARPES. A chemical potential shift of >150 meV was observed for the highest pump fluences in the TI phase after ultrafast laser excitation, which slowly rises and peaks after ∼0.6 ps, that persists for ∼8 ps. The slow rise and fall times are likely related to the partial relaxation of the distorted lattice order in the TI phase after laser excitation, and an associated change in electronic order. In contrast, no distinct excited state is observed in the TCI phase after photoexcitation, which we attribute to the low density of states and phase space available near the Fermi level. Our results demonstrate how excitation by ultrafast light pulses can probe the excited states and interactions in phase-rich topological materials.

## MATERIALS AND METHODS

Material: The flux method was employed to grow single crystals of NaCd_4_As_3_. Na, Cd, and As were mixed in a ratio of 1:8:3 and placed in an alumina crucible, sealed in a quartz tube for heating. The details of the sample preparation and characterization are discussed elsewhere.^31^

Time- and angle-resolved photoemission spectroscopy (tr-ARPES): An amplified Ti:sapphire laser was used to generate the pump and probe pulses for this experiment, operating at a central wavelength of 786 nm (1.58 eV), with ∼40 fs pulse duration and 10 KHz repetition rate. The fundamental wavelength was frequency doubled to 393 nm, which was then used to generate the seventh-order harmonic at a photon energy of 22.10 eV, ∼15 fs pulse duration, via high harmonic generation (HHG) in Kr gas. The energy resolution is close to 130 meV, limited by the bandwidth of the ultrashort EUV pulses. This HHG pulse was then used to photoemit electrons after excitation of the material by the 786 nm pump pulse. The details of the HHG source and the time- and angle-resolved resolved photoelectron spectroscopy setup can be found in our previous reports.^20,21,48–50^ Further details on determining *E*_F_ and temporal overlap are discussed in Sec. S6 of the supplementary material.^51^ The photoemitted electron intensity and kinetic energy at various angles were measured using a hemispherical electron analyzer, SPEC PHOIBOS 100. Prior to this measurement, the single crystal sample was cleaved in UHV vacuum better than 3 × 10^−10^ Torr and measured at various temperatures, primarily at 78 and 240 K, to access the TI and TCI phases of the material, respectively. The tr-ARPES spectra degraded over a period of ∼5–8 h for several samples measured—likely due to surface contamination from the presence of reactive Na atoms. To avoid any variation resulting from different samples and the limitation imposed by sample surface degradation over time, a controlled time-resolved measurement for a few representative fluences was done on the same sample to extract ΔT_e_. A similar procedure was used to determine the chemical potential shift, and additional fluence measurements were made by the measuring spectra at two delay points—at a negative time delay (before the pump pulse) and at 600 fs. The absorbed pump fluence in the TI (low temp.) phase is similar to the TCI (room temp.) phase for two reasons. First, space charge effects during the measurement start to appear at similar pump fluences for both the TI and TCI phases. Second, for NaCd_4_P_3_, a material from the same family as NaCd_4_As_3_, its absorption coefficient is reported to be similar at 298 and 130 K.^33^ The absorption coefficient of NaCd_4_P_3_ at room temperature is similar to the TCI phase of NaCd_4_As_3_.

## Data Availability

The data that support the findings of this study are available within the article and its supplementary material.
